# Angular Transmittance Analysis of a Novel Thermotropic Material

**DOI:** 10.1155/2013/538164

**Published:** 2013-11-21

**Authors:** Jian Yao

**Affiliations:** Faculty of Architectural, Civil Engineering and Environment, Ningbo University, Ningbo 315211, China

## Abstract

This paper uses inverse adding-doubling (IAD) method and Monte Carlo method for the simulation of the spectral angular transmittance of a novel kind of thermotropic material at different temperatures. The results show that the collimated light takes the major part at the beginning of the switching process and the scattered light is negligible. However, the scattered light increased to high above 80% of the total transmitted light with the largest angle distribution of scattered light about 30 degrees as temperature elevated.

Solar heat gain through a window is 20 times higher than through the neighbouring wall in summer [1], which causes overheating problems and high cooling energy consumption as well as glare problems. Windows are thus identified as an important area for energy-efficiency improvements. Therefore, windows with solar control function should be used for solar shading such as Low-e windows and thermotropic windows. In our previous work [2] a novel kind of thermotropic material was developed for energy efficient windows, and the radiation transmittance measurement showed that it has a better performance than Low-e double-glazed windows. As this kind of material scatters light when it reaches its LCST as shown in Figure 1, it is very significant to determine its angular transmittance, which is a key factor in affecting a window's energy performance [3]. However, a variety of previous researches about thermotropic materials are focused either on the switching temperature [4–6] and materials selection [7, 8] or on the total transmittance [9, 10]; no research, to our knowledge, has been conducted on the angular transmittance of thermotropic materials. Montgomery used an experimental system for angle-dependent light-scattering measurements with a movable photodetector [11]; however, this system used a He-Ne laser that did not cover the desirable spectrum of solar radiation (300–1800 nm). Currently, available spectroscopies such as UV3600 and lambda 950 are also unable to be used for such measurements. Thus, there is a need for a theoretical method to solve this problem. Monte Carlo for multilayered media (MCML) addresses this need as is a steady-state Monte Carlo simulation program for multilayered turbid media in which each layer has its own optical properties of absorption, scattering, anisotropy, and refractive index, and has been verified with other theories [12].

The optical properties of a turbid medium are characterized by the absorption coefficient (*μ*_*a*_), the scattering coefficient (*μ*_*s*_), and the anisotropy coefficient (*g*) [13]. Inverse adding-doubling is a technique developed by Prahl [14] that uses adding-doubling to figure out the optical properties of material from the observed  *μ*_*a*_,  *μ*_*s*_, and  *g*.

This method is called inverse adding-doubling (IAD): “inverse” to imply a reversal of the usual process of calculating reflection and transmission from optical properties and “adding-doubling” to indicate the method used to solve the radiative transport equation. The IAD algorithm and theory are described in Pickering's paper [15]. This paper adopted IAD method to determine the optical properties of the thermotropic material, and its results were used as the inputs of Monte Carlo simulation.

The structure for the sample of the thermotropic material, which is sandwiched in two glasses, is a three-layer structure, and the thickness of each layer is 5, 2, 5 mm. The refractive index coefficients of the glass and thermotropic layer are 1.52 and 1.35, respectively.

An ultraviolet/visible/near-infrared spectroscopy system (UV-3600, UV-Vis-NIR, SHIMADZU Corp.) equipped with a temperature controller was employed for spectral transmittance, reflectance, and collimated transmittance measurements. Then, these results were used as inputs of IAD for the simulation of the absorption coefficient *μ*_*a*_, the scattering coefficient *μ*_*s*_, and the anisotropy coefficient *g*.

The inputs including the absorption coefficient (*μ*_*a*_), the scattering coefficient (*μ*_*s*_) and the anisotropy coefficient (*g*) of the thermotropic material, using the inverse adding-doubling (IAD) method for the simulation of the angle-dependent light transmittance of thermotropic material at different temperatures at the wavelength range of 350–1800 nm, have been determined in Figures 2, 3, and 4.

It can be seen from Figures 2, 3, and 4 that the absorption coefficient (*μ*_*a*_) and the scattering coefficient (*μ*_*s*_) of the thermotropic material increased as the temperature rose, indicating that the scattering and absorption properties were strengthened as the material switched to turbid state. The anisotropy coefficients (*g*) of the thermotropic material at these temperatures kept below zero and decreased as temperature increased. It means that the back-scattering effect became more and more obvious. Then, these optical properties were used to perform the spectral angular transmittance simulation by MCML, and the results are shown in Figures 5, 6, and 7.

Figures 5–7 illustrate the light propagation in the thermotropic material at different temperatures at the wavelength of 500 nm. The light propagation at other wavelengths has similar phenomena and thus is not shown in this paper. The term  *Fzr*  (*z* and *r* are the thickness and width of the thermotropic material, resp.) means the light density distribution in the thermotropic material. It can be seen that the scattered light increased as temperature rose, while the collimated light decreased. At temperature 32°C, the scattered light is much less than collimated light as the material is beginning to be turbid; however, when the temperature increased to 36°C, the scattered light is comparable with collimated light as the material is totally turbid. However, these figures can only give the light-scattering property of such material and the light propagation in it. In order to determine the angular transmittance distribution, the orientation of transmitted light at the interface of number 4, as shown in Figure 1, was recorded and depicted in Figures 8, 9, and 10.

According to Figures 8–10, it was found that the spectral angular transmittance distribution of the thermotropic material at interface no. 4 at 32°C was mainly between 0 and 5 degrees, indicating that the collimated light was the major part (high above 95%) and less light was scattered by the material. The scattered light was between 0 and 50 degrees due to the Snell's law [16]. As the temperature increased to 34°C, the percentage of scattered light increased in all the angles of transmittance and collimated light dropped significantly. The collimated light in the wavelength range of 900–1300 nm was still the most part of transmitted light, which (about 85–90%) is stronger than that of others (about 60–75%). This phenomenon was occurred as the temperature rose to 34°C. The reason for it may be attributed to the strong light absorption property of water in this wavelength range, which affects the scattering property of the thermotropic material. At the temperature of 36°C, the spectral transmittance in the degrees of 0–5 dropped to below 20% except the wavelength range of 900–1300 nm, and the scattered light increased to high above 80% of the total transmitted light with the largest angle distribution of scattered light about 30 degrees. In other words, the scattering light cannot be ignored as it has become the major part, and therefore, it is very significant for the solar heat gain calculation of thermotropic windows.

In this paper, we used IAD method for the absorption coefficient (*μ*_*a*_), the scattering coefficient (*μ*_*s*_) and the anisotropy coefficient (*g*) of the thermotropic material, and propose a Monte-Carlo method for the simulation of the spectral angular transmittance of thermotropic material at different temperatures. The results show that the collimated light takes the major part at the beginning of switching process and the scattered light is negligible. However the scattered light increased as temperature rose and the spectral transmittance in the degree of 0–5 dropped to below 20% of the total transmitted light except the wavelength range of 900–1300 nm, and the scattered light increased to high above 80% of the total transmitted light with the largest angle distribution of scattered light about 30 degree. As a conclusion, Monte Carlo simulation (MCML) is an effective method for the determination of angle-dependent light transmittance of thermotropic material, and the results of these simulations can be used to calculate the solar heat gain for thermotropic windows.

## Figures and Tables

**Figure 1:**
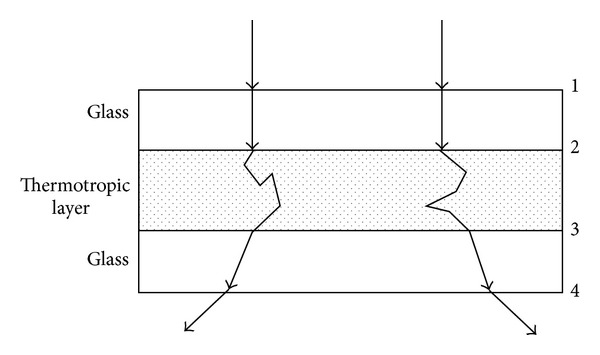
The three-layer structure of a thermotropic window.

**Figure 2:**
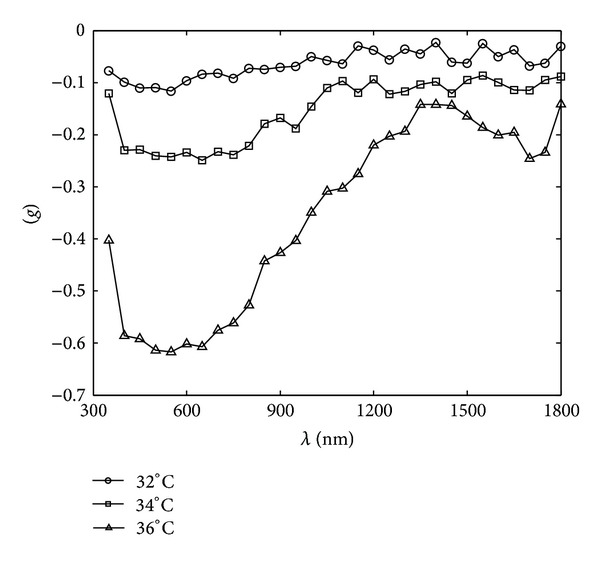
The spectral absorption coefficients of the thermotropic material at 32, 34, and 36°C, respectively.

**Figure 3:**
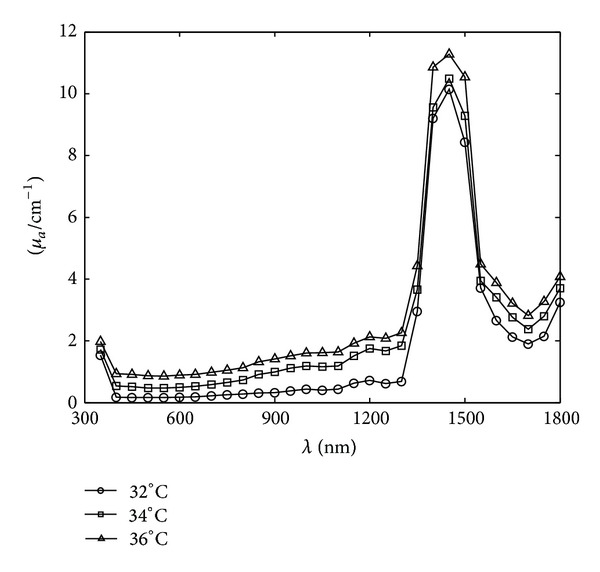
The spectral scattering coefficients of the thermotropic material at 32, 34, and 36°C, respectively.

**Figure 4:**
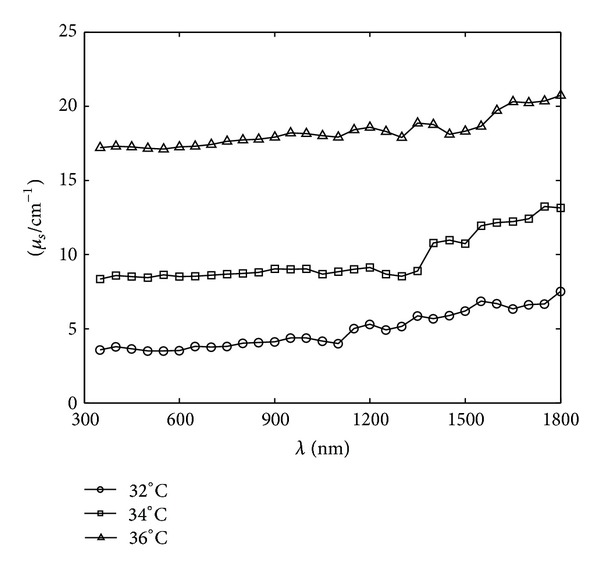
The spectral anisotropy coefficients of the thermotropic material at 32, 34, and 36°C, respectively.

**Figure 5:**
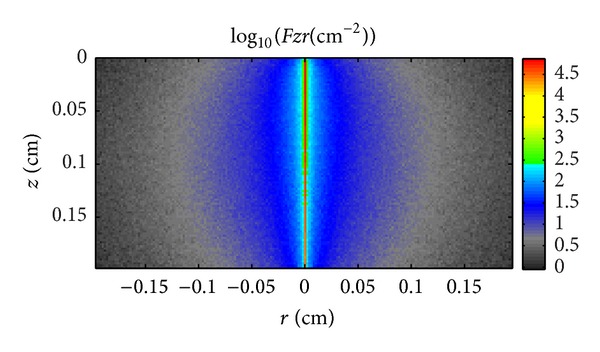
Light propagation in the thermotropic material at 32°C at the wavelength of 500 nm.

**Figure 6:**
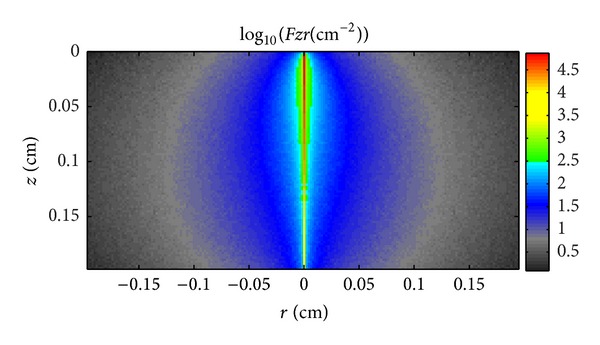
Light propagation in the thermotropic material at 34°C at the wavelength of 500 nm.

**Figure 7:**
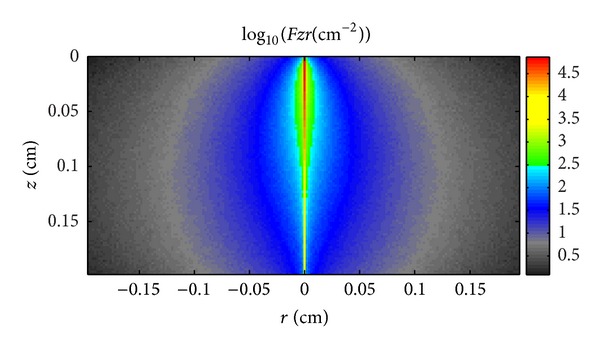
Light propagation in the thermotropic material at 36°C at the wavelength of 500 nm.

**Figure 8:**
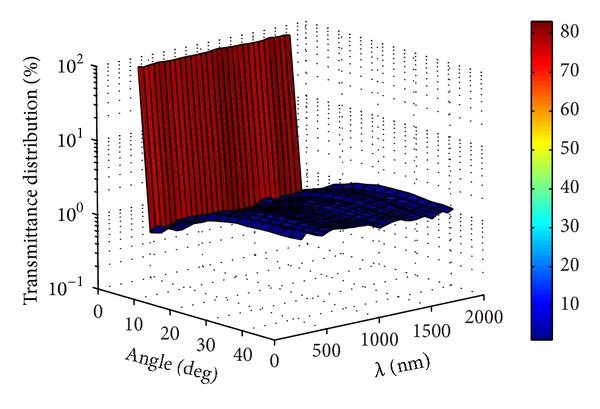
Spectral angular transmittance distribution of the thermotropic material at 32°C.

**Figure 9:**
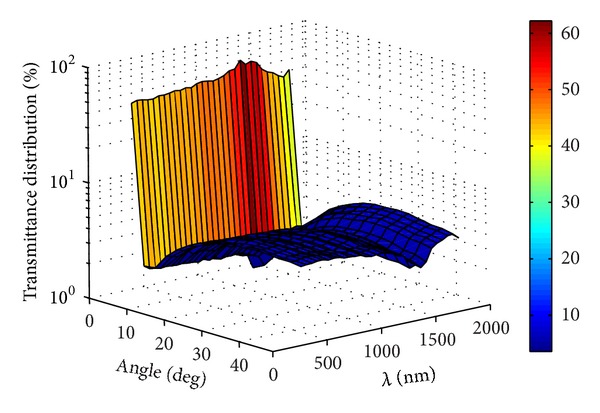
Spectral angular transmittance distribution of the thermotropic material at 34°C.

**Figure 10:**
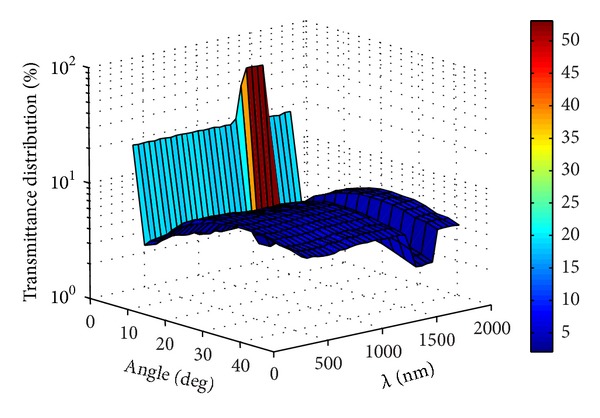
Spectral angular transmittance distribution of the thermotropic material at 36°C.
